# Construction Notes

**Published:** 1905-12-16

**Authors:** 


### CONSTRUCTION NOTES.

THE Selby Joint Isolation Hospital, which has been built at a cost of about £6,500, has been formally opened. There are two complete blocks; one for typhoid and diphtheria, and another for scarlet fever. The drainage is on the septic tank system.

ON the 4th inst. a new isolation hospital was opened at Melton Mowbray. The buildings, which cost nearly £9,000, comprise distinct accommodation for fever and isolation cases, and an administrative block. The plans were prepared by Mr. E. Jeenes.

THE South Staffordshire Joint Small-pox Hospital, which has been erected at Bradley, at a cost, including site, of about £18,000, was formally opened on the 4th inst. The plans were prepared by Mr. George Green, Borough Surveyor, Wolverhampton.

A NEW workhouse infirmary was opened on the 7th inst. at Stepping Hill, Stockport. Accommodation is provided for 340 patients, and the nurses' home which is attached will accommodate 36 nurses and 24 officials and servants. The estimated cost per bed is £130. Mr. W. H. Ward, of Birmingham, is the architect.

THE new buildings of the Harrogate and Knaresborough Joint Isolation Hospital, at Thistle Hill, near Knaresborough have been opened. Separate blocks are provided for scarlet fever, typhoid, diphtheria, isolation, and administration, and occupy 10½ acres, but as the entire site is about 24 acres there is ample room for extension.

A NEW Isolation Hospital was opened at Rotherham on the 7th inst. It is excellently situated on an eminence a mile and a half away from the town. The hospital includes scarlet fever, typhoid, and diphtheria pavilions and an administrative block, and the cost of building, furnishing, and site is £19,285. A bacteriological laboratory forms an important addition to the building. The architect is Mr. J. Platts.

